# Prevention and care of hepatitis B in the rural region of Fatick in Senegal: a healthcare workers’ perspective using a mixed methods approach

**DOI:** 10.1186/s12913-019-4416-3

**Published:** 2019-09-04

**Authors:** Tchadine Djaogol, Marion Coste, Fabienne Marcellin, Antoine Jaquet, Fanny Chabrol, Tamara Giles-Vernick, Aldiouma Diallo, Maria Patrizia Carrieri, Sylvie Boyer, for the ANRS 12356 AmBASS Study Group, Cyril Bérenger, Marwan al Qays Bousmah, Sylvie Boyer, Patrizia Carrieri, Marion Coste, Maëlle de Sèze, Tchadine Djaogol, Gwenaëlle Maradan, Fabienne Marcellin, Carole Treibich, Elhadji Ba, Aldiouma Diallo, Fambaye Dièye, Assane Diouf, Elhadji Bilal Faye, Assane Ndiaye, Lauren Perieres, Cheikh Sokhna, Coumba Touré Kane, Gora Lo, Anna Julienne Selbé Ndiaye, Philippe Halfon, Sofiane Mohamed, Nicolas Rouveau, Maria-Camila Calvo Cortès, Gabrièle Laborde-Balen

**Affiliations:** 1 Aix Marseille Univ, INSERM, IRD, SESSTIM, Sciences Economiques & Sociales de la Santé & Traitement de l’Information Médicale, Marseille, France; 2 ORS PACA, Observatoire régional de la santé Provence-Alpes-Côte d’Azur, Marseille, France; 3 INSERM U1219, ISPED, Université de Bordeaux, Bordeaux, France; 4 Centre Population et Développement (CEPED), French Institute for Research on Sustainable Development (IRD), Université de Paris, INSERM SAGESUD, Paris, France; 5 Emerging Diseases Epidemiology Unit, Pasteur Institute, Paris, France; 6 VITROME UMR 257 Institut de Recherche Pour le Développement, Dakar, Senegal

**Keywords:** Hepatitis B, HBV, Mixed-methods, Healthcare workers, Prevention, Vaccination at birth, Senegal, Africa, Vertical transmission, Mother-to-child transmission, Decentralized care

## Abstract

**Background:**

In countries where hepatitis B virus (HBV) is endemic, including Senegal, the World Health Organization recommends systematic HBV screening of pregnant women and vaccination at birth to prevent mother-to-child transmission (MTCT). This study investigated healthcare workers’ (HCW) knowledge and practices regarding HBV prevention and care in the rural region of Fatick in Senegal, as well as challenges they faced in implementing prevention activities related to HBV MTCT.

**Methods:**

A mixed-methods survey was conducted between May–July 2017 among 112 HCW working in 15 healthcare facilities in two districts of the Fatick region using face-to-face questionnaires and semi-structured interviews. Descriptive statistics and chi-square/Mann-Whitney tests were used to analyze quantitative data, while qualitative data were analyzed thematically.

**Results:**

The study population included 87 HCW in the quantitative component (83% women, median age [interquartile range, IQR] = 35 [31–40] years) and 11 in the qualitative component. A knowledge gap was observed in key areas of HBV infection: only 24, 51 and 38%, respectively, correctly reported that early HBV acquisition is associated with a high risk of developing chronic infection, that perinatal transmission is one of the main modes of HBV transmission in Senegal, and that three to four doses of HBV vaccine are required to ensure immunization in children. Despite good acceptability of systematic screening of pregnant women and vaccination at birth, only 48% of HCW mainly involved in prenatal care and 71% of those involved exclusively in vaccination routinely performed these two key interventions. HCW reported several structural barriers that may hinder their implementation: a lack of training in HBV and in counseling, poor availability of rapid diagnostic tests (RDT), high costs of both screening and treatment, a lack of adequate information on treatment options and missed opportunities for vaccination at birth.

**Conclusions:**

HCW working in the Fatick region may be insufficiently trained and supported to effectively implement HBV prevention strategies. Our findings suggest an urgent need to strengthen MTCT prevention in this region, by improving HCW knowledge in key areas of HBV infection, providing RDT and antiviral treatment at low cost, and enhancing community-based interventions for the timely vaccination of newborns.

**Electronic supplementary material:**

The online version of this article (10.1186/s12913-019-4416-3) contains supplementary material, which is available to authorized users.

## Background

Among the 257 million people chronically infected by hepatitis B virus (HBV) worldwide, 60 million are living in sub-Saharan Africa, the second most affected region in the world after Asia [1, 2]. HBV prevalence is especially high in West Africa with rates estimated between 7 and 18% in the adult population, depending on the country [3].

HVB prevention, and especially prevention of mother-to-child transmission (MTCT), is a high priority in this endemic region for three main reasons. First, perinatal (vertical) transmission and early childhood (horizontal) transmission are the main modes of HBV acquisition in this area [2, 4, 5]. Second, approximately 90% of children infected during their first year of life will develop chronic HBV infection (versus only 5% of people infected during adulthood). Third, compared with horizontal transmission and infection acquired during adulthood, vertical transmission is associated with a higher risk of serious liver complications later on in life including cirrhosis and hepatocellular carcinoma [2, 6, 7].

The World Health Organization (WHO) recommends the following three key interventions in all endemic areas: i) systematic screening of pregnant women, ii) vaccination of newborns within 24 h with HBV monovalent vaccine, followed by 2 to 3 additional vaccine doses, and iii) treatment of HBV-positive pregnant women using antiviral therapy based on tenofovir [2, 8, 9].

In Senegal, the prevalence of chronic HBV infection is estimated at 11% according to a recent systematic review [3]. HBV screening is not mandatory for pregnant women but is generally proposed during prenatal consultations. In addition to the pentavalent vaccine administrated to newborns at 6, 10 and 14 weeks after birth, in 2016 vaccination at birth (first dose) was introduced as part of the country’s expanded immunization program (EPI) [10]. A working group was also initiated in 2016 to develop national guidelines to promote access to HBV treatment - including access for pregnant women - at the decentralized level of the healthcare system. At the time of this study, however, these guidelines were not yet officially adopted, and access to antiviral treatment was only available in major hospitals located in the country’s two main cities, Dakar and St Louis.

Good adhesion to WHO recommendations and adequate training of healthcare workers (HCW) are essential to ensure successful prevention of HBV MTCT in Senegal, especially at the decentralized level of the healthcare system, where HCW are responsible for both pre- and post-natal care, including routine HBV screening of pregnant women, pre- and post-test counseling and HBV vaccination at birth.

Several studies conducted on physicians, medicine students and non-medical HCW working in urban areas in sub-Saharan African countries have highlighted suboptimal overall knowledge of HBV prevention and care and related work practices [11–15]. In Senegal, a recent study conducted among physicians in the major hospitals in Dakar and St. Louis also highlighted that these professionals lacked training in viral hepatitis management [16]. HBV prevention and care bring acute and complex challenges to HCW, especially in rural and decentralized areas where human and technical resources are scarce and where the population has limited financial resources and a low educational level [17, 18]. However, these challenges are greatly under-documented. Further studies on HCW working in decentralized and rural healthcare facilities are therefore required to investigate their adhesion to the WHO strategies introduced to reduce HBV vertical transmission, and to assess whether they are adequately trained and supported to implement these strategies effectively.

In this study, we aimed to investigate HBV prevention and care knowledge and practices of HCW working in two districts of the rural region of Fatick in Senegal, as well as challenges they faced to implement prevention activities related to HBV MTCT.

## Methods

### Study setting

Fatick is one of the 14 regions of the Senegal with 714,389 inhabitants. It is located 132 km east of Dakar, at the border with Gambia. The region is mostly rural with agricultural, livestock farming and fishing being its main resources. Maternal and child health indicators in the Fatick region are among the best in the country. In 2014, coverage for the pentavalent vaccine was 84% [19]. Furthermore, the region ranked third in the country for infant and child mortality rates (estimated at 52 and 73 per 1000, respectively) and fourth for maternal mortality rate (estimated at 365 per 1000) [20]. The Fatick region is divided into seven health districts with 110 healthcare facilities, including the regional hospital, 7 healthcare centers that constitute reference healthcare facilities for each district, and 102 primary healthcare posts which are first-contact facilities offering basic services including maternal and child healthcare as well as normal deliveries. The present study was conducted in two of the seven health districts: i) the Fatick district which includes the regional capital city of Fatick and has both the largest district-level population and the best healthcare infrastructure in the region and ii) the Niakhar district, which is a typical rural district of the region with limited health infrastructure and human resources. Together, both selected districts account for 37 of the 110 health facilities in the region (i.e. 34%) and 274 of 597 HCW (i.e. 46%). Besides the regional hospital located in the capital, the 37 health facilities of the two districts comprise *2* healthcare centers and 34 primary healthcare posts.

This study area was chosen for the two following main reasons. First, the present study is nested within the ongoing research project AmBASS (ANRS 12356) which is being implemented in the area covered by the Niakhar Health and Demographic Surveillance System located in the Niakhar and Fatick districts. The AmBASS project is a large population-based survey which aims to study the epidemiology, and socioeconomic and public health impacts of HBV chronic infection in rural area in Senegal [21]. Second, the Fatick region has been chosen by the Senegalese National Hepatitis Program as a pilot region for the decentralization of HBV care and treatment, which makes this region particularly interesting given our study objectives [22].

### Study design

We used a cross-sectional mixed-methods survey: the quantitative component sought to document both HCW global knowledge of HBV and HCW acceptability of interventions for prevention of MTCT (including screening of pregnant women and vaccination at birth), while the qualitative component explored HCW perceptions and field experience regarding these interventions, as well as the daily challenges they faced in implementing them.

The sample size for the quantitative component was optimized (*n* = 100) to both provide descriptive statistics and to take into account human resource and time constraints.

Two-stage reasoned sampling was used for the quantitative component of the survey as follows: in the first stage, we selected the healthcare facilities to include in the survey from the 37 healthcare facilities located in the Niakhar and Fatick districts. To do this, a purposive approach was adopted using the following selection criteria in order to capture the diversity of the healthcare supply: location of the facility (urban versus rural area), level of decentralization (regional hospital, district healthcare center or primary healthcare post), and number of HCW working in each facility (< 10 versus ≥ 10). Accordingly, 15 facilities were selected as follows: the Fatick regional public hospital which has the largest maternity unit in the region, the 2 healthcare centers of the two districts (both public) and 12 primary healthcare posts (10 public and 2 private) (See Additional file 1). In the second stage, all eligible HCW in the 15 healthcare facilities chosen at first stage were invited to participate in the survey. Eligibility criteria were based on HCW roles and daily activities in the facilities. In the 12 primary healthcare posts, all HCW were eligible to participate in the survey as they had overlapping roles and duties given the small staffing numbers (< 10). In the two healthcare centers and in the regional hospital, where staff had better-defined roles and duties, only HCW in charge of general consultation, vaccination, pre- and post-natal consultations and deliveries were eligible.

The qualitative component of the survey was proposed to willing and available HCW who were eligible for the quantitative survey in the 15 selected facilities and whose characteristics represented all the categories of HCW participating in the quantitative survey. However, to avoid potential response bias, HCW could only participate in one component of the study (i.e. either in the qualitative or quantitative component). We also included one HCW working in a very well-known traditional health center which plays a key role in the care of the population living in the study area.

### Data collection

Data collection was conducted between May 24th and July 6th 2017. Quantitative data on HCW characteristics were collected through face-to-face paper-based questionnaires structured around the following modules: i) socio-demographic characteristics, ii) training, experience and activities in the field of HBV, iii) knowledge about HBV natural history, epidemiology, screening, vaccination and treatment, and iv) acceptability and perception of MTCT prevention strategies (See Additional file 2). Most questions had only three possible answers (*“yes”, “no”* and *“I do not know”*). Multi-modal knowledge questions with one correct answer and several incorrect answers were also asked.

Qualitative data were collected using in-depth individual interviews conducted by a Master’s degree student in international public health. The following themes were explored during semi-structured interviews: knowledge and representations of HCW about HBV infection (especially its transmission modes), experience in HBV screening, vaccination, treatment, and challenges faced in the implementation of these activities. The principle of data saturation was respected [23].

Questionnaire administration lasted on average 30 min, and qualitative interviews between 19 and 52 min. Quantitative data were collected mainly in French, the official language in Senegal. However, a local experienced translator helped translate questionnaires into Serer for HCW who had a low level of French (approximately 20 people). All qualitative interviews were conducted in French and audio-recorded with the participants’ consent.

### Data analysis

The study population of the quantitative component included HCW involved in activities related to maternal and child health. They were selected using seven questions documenting whether or not their routine work practice involved the following activities: i) prenatal care, ii) post-natal care, iii) deliveries and iv) vaccination. According to their answers to these questions, HCW were classified into two groups:

1. HCW mainly involved in prenatal care activities, i.e. those who reported they performed only prenatal consultations, and those who reported both prenatal consultations and at least one other activity from the following: postnatal care, deliveries or vaccination (Group 1);
2. HCW only involved in vaccination activities (Group 2).

Quantitative data on HCW knowledge, perceptions and practices concerning HBV were first described overall and then compared between the two groups of HCW using a Chi-square or Fisher’s exact test for categorical variables, and Mann-Whitney test for continuous variables. To this end, binary knowledge variables were built by grouping correct answers against incorrect answers (including the “*I do not know*” answers) (Table 1). As there were few missing data (< 5%), descriptive analyses (number (%) and median [interquartile range, IQR]) were performed on complete data. R software (version 3.4.2) was used for the analyses.

**Table 1:** Description of the knowledge variables and definition of correct answers

Knowledge questions	Possible answers	Correct answer^a^
* General knowledge of HBV (natural history and epidemiology)*		
What kind of infectious agent causes hepatitis B?	Virus	Virus
	Bacterium	
	I do not know	
What organ is affected by HBV?	Liver	Liver
	Lung	
	Stomach	
	Heart	
	I do not know	
What complications can be caused by hepatitis B?	Liver cancer (*yes; no; I do not know*)	Yes
	Cirrhosis (*yes; no; I do not know*)	Yes
What are the modes of HBVtransmission?	Dust (*yes; no; I do not know*)	No
	Contaminated water (*yes; no; I do not know*)	No
	Perinatal transmission (from mother to child) (*yes; no; I do not know*)	Yes
	Breastfeeding (*yes; no; I do not know*)	No
	Horizontal transmission (during childhood through contacts with infected blood) (*yes; no; I do not know*)	Yes
	Percutaneous transmission or transmission through mucosae (*yes; no; I do not know*)	Yes
	Sexual transmission (*yes; no; I do not know*)	Yes
Is perinatal transmission (from mother-to-child) one of the main modes of transmission in Senegal?	Yes	Yes
	No	
	I do not know	
Are people infected by HBV duringchildhood (< 1 year) going to developchronic infection?	Yes, more than 80% of them	Yes, more than 80% of them
	Yes, approximately 50% of them	
	Yes, only 5%	
	I do not know	
* Specific knowledge on HBV screening*		
What are the priority groups for whichroutine screening of chronic hepatitis B is recommended?	Pregnant women (*yes; no; I do not know*)	Yes
	Blood donors (*yes; no; I do not know*)	Yes
	Health Care Workers (*yes; no; I do not know*)	Yes
	HIV-positive patients (*yes; no; I do not know*)	Yes
What kind of test can be used to screen for HBV in adults and children > 1 year	Rapid diagnostic test (*yes; no; I do not know*)	Yes
	Laboratory-based immunoassay (*yes; no; I do not know*)	Yes
* Specific knowledge on HBV vaccination*		
What are the potential strategies for reducing perinatal transmission?	Vaccination at birth (*yes; no; I do not know*)	Yes
	Antiviral treatment for the mother (*yes; no; I do not know*)	Yes
	Immune globulin administration (*yes; no; I do not know*)	Yes
When should the first dose of HBV vaccine be administered to children?	Within 24 h after birth	Within 24 h after birth
	Within the first week of life	
	At 6 weeks	
	At 1 year old	
	I do not know	
In total, how many injections arerequired to immunize children against hepatitis B infection?	1 injection	3 or 4 injections
	2 injections	
	3 injections	
	4 injections	
	Other	
	I do not know	
* Specific knowledge on HBV treatment*		
Is there an effective treatment against chronic hepatitis B infection?	Yes	Yes
	No	
	I do not know	
Which of the following treatments has proven efficacy against chronic hepatitis B infection?	Lamivudine (*yes; no; I do not know*)	Yes
	Tenofovir (*yes; no; I do not know*)	Yes
	Traditional treatment (*yes; no; I do not know*)	No
How long does antiviral therapy against chronic hepatitis B infection continue?	1 week	For life
	A month	
	For life	
	I do not know	

*HBV* hepatitis B virus

^a^ All answers other than the correct one were recoded as incorrect

Qualitative data analysis was based on grounded theory, which is an inductive research method suitable for the exploration of health care experience [24]. Recorded interviews were first transcribed and then analyzed using sequenced thematic coding, as described by Paillé and al [25].

## Results

### Study population

**Fig. 1:**
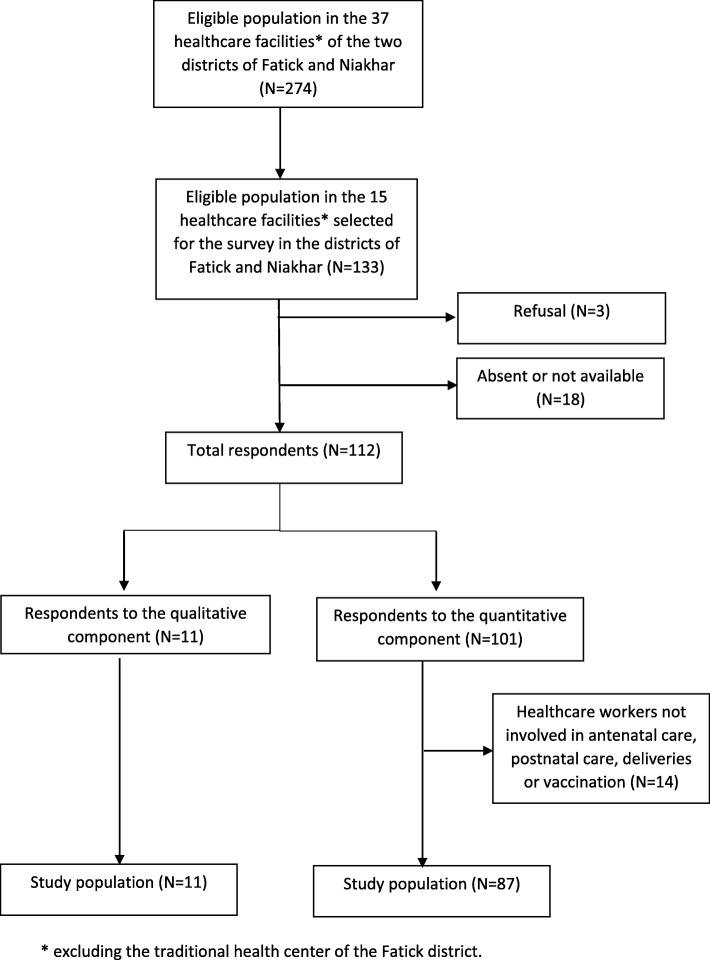
Selection of the study population, *n* = 98

Of the 133 eligible HCW, 112 participated in the survey (101 and 11 in the quantitative and qualitative components, respectively), i.e. a global participation rate of 84%. Reasons for non-participation included absence or unavailability at the time of the survey (*n* = 18, 14%) and refusal to participate (*n* = 3, 2%). Among the 101 participants in the quantitative component, based on their questionnaire answers, we excluded 14 HCW who reported not being involved in prenatal or postnatal care, deliveries or vaccination (including 3 physicians) (Fig. 1). Accordingly, the study population in the quantitative component comprised 87 HCW. Its main characteristics are described in Table 2.

**Table 2:** Characteristics of healthcare workers who participated in the quantitative survey (*n* = 87)

Variable (% of missing data)	Total	HCW mainly involved in prenatal care activities (Group 1)	HCW involved exclusively in vaccination activities (Group 2)	*p*-value^a^
	(*N* = 87)	(*N* = 47, 54%)	(*N* = 40, 46%)	
	N (%) or median [IQR]			
Age (years) (4%)	35 [30–40]	32 [27–37]	37 [33–50]	0.006
Gender				
Male	15 (17)	6 (13)	9 (22)	
Female	72 (83)	41(87)	31(78)	0.231
Education level				
≤ middle school	41 (47)	12 (25)	29 (72)	
> middle school	46 (53)	35 (75)	11 (28)	< 10^−3^
Initial training in the field of health or social care				
Nurses (registered and licensed professional)	25 (29)	16 (34)	9 (22.5)	< 10^−3^
Nursing assistant	17 (19)	7 (15)	10 (25)	
Midwife	15 (17)	15 (32)	0 (0)	
Laboratory technician	1 (1)	0	1 (2.5)	
Community healthcare worker	29 (33)	9 (19)	20 (50)	
Professional experience (years) (1%)	7 [4–14]	5 [3–10]	11 [7–17]	0.001
Decentralization level of the healthcare facility				
Regional hospital	8 (9)	7 (15)	1 (2)	0.150
District healthcare center	26 (30)	13 (28)	13 (33)	
Primary healthcare post	53 (61)	27 (57)	26 (65)	

*HCW* Healthcare Workers, *HBV* hepatitis B virus, *IQR* interquartile range

^a^ Chi-square test or Fisher’s exact test for categorical variables and Mann-Whitney test for continuous variables

The majority were women (83%) and median [IQR] age was 35 [30–40] years. More than half (53%) had an education level higher than middle school. Approximately one third (33%) were community healthcare workers, 29% registered or licensed professional nurses, 19% nursing assistants, 17% midwives and 1% laboratory technicians. The median [IQR] duration of HCW professional experience was 7 [4–14] years. A majority (61%) worked in primary healthcare posts, 30% in the two district healthcare centers and 9% in the regional hospital.

Forty-seven (54%) HCW were mainly involved in prenatal care (Group 1) and 40 (46%) exclusively in vaccination (Group 2). HCW were older in Group 2 than in Group 1 (37 [33–50] versus (32 [27–37]), *p* = 0.006). They also had a lower educational level and were less qualified: only 28% had an educational level higher than middle school (versus 75% in Group 1, *p* < 10^− 3^) and most (75%) were community HCW or nursing assistants (versus 52% in Group 1, p < 10^− 3^).

Of the 11 HCW included in the qualitative component, 3 were physicians, 4 were nurses, 2 were midwives, 1 was a community healthcare worker and 1 was a laboratory technician. Six men and five women participated (Table 3).

**Table 3:** Characteristics of healthcare workers who participated in the qualitative survey (*n* = 11)

Individual interview n^°^	Initial training	Healthcare facility type	Activity
1	Physician	Regional hospital	Medical consultation
2	Physician	Healthcare center	Medical consultation
3	Physician	Regional hospital	Medical consultation
4	Nurse	Primary healthcare post	Prenatal care activities, routine consultation, immunization
5	Midwife	Primary healthcare post	Prenatal care activities, deliveries, immunization
6	Midwife	Primary healthcare post	Prenatal care activities, deliveries
7	Nurse	Healthcare center	Prenatal care activities, routine consultations, deliveries
8	Community healthcare worker	Primary healthcare post	Prenatal care activities, routine consultation, immunization
9	Nurse	Primary healthcare post	Prenatal care activities, routine consultations, deliveries
10	Laboratory technician	Traditional health center	Head of the traditional health center
11	Nurse	Regional hospital	Routine consultations

### HCW general knowledge of hepatitis B

#### Natural history and epidemiology

Overall, 81% of participating HCW were aware that a virus causes HBV, 94% knew that HBV affects the liver, and 80 and 85% knew that complications of HBV include cirrhosis and liver cancer, respectively (Table 4).

**Table 4:** General knowledge of HBV (natural history and epidemiology) among healthcare workers participating in the quantitative component of the survey (*n* = 87)

Variables (% of missing data)	Total	HCW mainly involved in prenatal care activities (Group 1)	HCW involved exclusively in vaccination activities (Group 2)	*p*-value^a^
	(*N* = 87)	(*N* = 47, 54%)	(*N* = 40, 46%)	
	N (%)			
What kind of infectious agent causes hepatitis B? (1%)				
*Correct answer*	70 (81)	40 (87)	30 (75)	
*Incorrect answer*	16 (19)	6 (13)	10 (25)	0.15
What organ is affected by HBV? (8%)				
*Correct answer*	75 (94)	43 (98)	32 (89)	
*Incorrect answer*	5 (6)	1 (2)	4 (11)	0.12
What complications can be caused by hepatitis B?				
Liver cancer				
*Correct answer*	74 (85)	43 (91)	31 (78)	0.07
*Incorrect answer*	13 (15)	4 (9)	9 (22)	
Cirrhosis (1%)				
*Correct answer*	69 (80)	40 (87)	29 (72)	
*Incorrect answer*	17 (20)	6 (13)	11 (28)	0.09
What are the modes of HBV transmission?				
Dust (5%)				
*Correct answer*	68 (82)	37 (80)	31 (84)	0.69
*Incorrect answer*	15 (18)	9 (20)	6 (16)	
Contaminated water (1%)				
*Correct answer*	61 (71)	34 (72)	27 (69)	0.75
*Incorrect answer*	25 (29)	13 (28)	12 (31)	
Perinatal transmission				
*Correct answer*	76 (87)	41 (87)	35 (87)	0.65
*Incorrect answer*	11 (13)	6 (13)	5 (13)	
Breastfeeding (1%)				
*Correct answer*	41 (48)	22 (47)	19 (49)	0.86
*Incorrect answer*	45 (52)	25 (53)	20 (51)	
Horizontal transmission				
*Correct answer*	67 (77)	40 (85)	27 (67)	0.05
*Incorrect answer*	20 (23)	7 (15)	13 (33)	
Percutaneous transmission or transmission through mucosae (2%)				
*Correct answer*	37 (44)	25 (53)	12 (32)	0.05
*Incorrect answer*	48 (56)	22 (47)	26 (68)	
Sexual transmission				
*Correct answer*	52 (60)	31 (66)	21 (52)	0.20
*Incorrect answer*	35 (40)	16 (34)	19 (48)	
Is perinatal transmission (from mother-to-child) one of the main modes of transmission in Senegal? (1%)				
*Correct answer*	44 (51)	28 (60)	16 (41)	
*Incorrect answer*	42 (49)	19 (40)	23 (59)	0.09
Are people infected by HBV during childhood (< 1 year) going to develop chronic infection? (2%)				
*Correct answer*	19 (24)	13 (29)	6 (15)	
* Incorrect answer*	66 (76)	32 (71)	34 (85)	0.12

*HCW* Healthcare workers, *HBV* hepatitis B virus

^a^ Chi-square test or Fisher’s exact test

However, qualitative interviews showed that this basic knowledge was not always well understood. More specifically, the causes of HBV were confused with other risk factors of liver disease such as alcohol or peanut consumption:

- *“The main cause of hepatitis B, in theory, is said to be alcoholism.” (Interview HCW 11)*
- *“Others also say that there are peanuts. In places where you consume a lot of peanuts, there is a risk [of hepatitis B]” (Interview HCW 11)*

Overall, 87% of HCW knew that HBV might be transmitted from mother to child during pregnancy and/or delivery (Table 4). However, other modes of transmission tended to be less correctly reported, especially in HCW involved in vaccination activities only (i.e., Group 2). More specifically, in this group, only 67, 52 and 32% of HCW knew that HBV may be transmitted through, respectively, horizontal transmission, sexual transmission and percutaneous or mucous membranes (versus 85, 66 and 53% in HCW mainly involved in prenatal care (i.e., Group 1), *p* = 0.05, *p* = 0.20 and *p* = 0.05, respectively) (Table 4). In addition, a substantial proportion of HCW reported incorrect HBV transmission modes: more than half (52%) reported that HBV could be transmitted through breastfeeding, 29% through contaminated water and 18% through dust.

Qualitative interviews also brought to light a large number of inaccurate beliefs about possible fecal-oral transmission, transmission from circulating air, and transmission from dirty hands.

- *“So there are the body fluids, fecal-oral transmission is possible, if I’m not mistaken.” (Interview HCW 1)*
- *“The modes of transmission, there’s mother-to-child, sexual, blood, through circulating air.” (Interview HCW 6)*
- *“There’s sexual (transmission), there are also the factors of, the factors of… what are they called, not hereditary but, what are they called, like natural.” (Interview HCW 11)*

Basic knowledge of the epidemiology of HBV was also low. Only 51% of HCW knew that perinatal transmission is one of the main modes of HBV transmission in Senegal and only 24% knew that early acquisition is associated with a high risk (> 80%) of developing chronic HBV infection (Table 4).

### Screening

#### Specific knowledge, skills and capacities for HBV screening

Overall, HCW in both groups had good knowledge of priority groups for HBV screening. A large majority listed pregnant women (92%), blood donors (87%) and healthcare workers (82%) but only (64%) reported HIV-positive patients (Table 5). Knowledge of HBV screening tests was relatively low. Only 70 and 46% knew that HBV could be screened using, respectively, rapid diagnostic tests (RDT) and laboratory-based immunoassays. HCW in Group 1 were significantly more likely to know that RDT can be used for HBV screening than HCW in Group 2 (81% versus 57%, *p* = 0.02).

**Table 5:** Knowledge, skills, capacity and acceptability of HBV screening among healthcare workers participating in the quantitative component of the survey (*n* = 87)

Variables (% of missing data)	Total	HCW mainly involved in prenatal care activities (Group 1)	HCW involved exclusively in vaccination activities (Group 2)	*p*-value^a^
	(*N* = 87)	(*N* = 47, 54%)	(*N* = 40, 46%)	
	N (%)			
*Specific knowledge, skills and training on testing and counseling*				
What are the priority groups for which routine screening of chronic hepatitis B is recommended?				
Pregnant women				
*Correct answer*	80 (92)	44 (94)	36 (90)	0.41
*Incorrect answer*	7 (8)	3 (6)	4 (10)	
Blood donors				
*Correct answer*	75 (87)	39 (83)	36 (90)	0.90
*Incorrect answer*	12 (13)	8 (17)	4 (10)	
HCW				
*Correct answer*	71 (82)	39 (83)	32(80)	0.72
*Incorrect answer*	16 (18)	8 (17)	8(20)	
HIV-positive patients				
*Correct answer*	56 (64)	32 (68)	24 (64)	0.43
*Incorrect answer*	31 (36)	15 (32)	16 (40)	
What kind of test can be used to test for HBV in adults and children > 1 year?				
Rapid diagnostic test				
*Correct answer*	61 (70)	38 (81)	23 (57)	0.02
*Incorrect answer*	26 (30)	9 (19)	17 (43)	
Laboratory-based immunoassay test (1%)				
*Correct answer*	40 (46)	25 (53)	15 (38)	0.17
*Incorrect answer*	46 (54)	22 (47)	24 (62)	
Have you already benefited from training in HBV?				
Yes (in my initial training, in continued education)	51 (59)	22 (47)	29 (72)	
No or I do not know	36 (41)	25 (53)	11 (28)	0.01
Have you already had training in counseling or therapeutic education for other diseases? (2%)				
Yes	57 (67)	25 (54)	32 (82)	
No or do not know	28 (33)	21 (46)	7 (18)	0.01
Have you already had training in counseling or therapeutic education for hepatitis B? (2%)				
Yes	17 (20)	11 (24)	6 (15)	
No or I do not know	68 (80)	35 (76)	33 (85)	0.33
Do you feel you are trained well enough to provide adequate counseling to HBV-positive patients?				
Yes (a lot, somewhat, a little)	63 (72)	38 (81)	25 (62)	
No (not at all, I do not know)	24 (28)	9 (19)	15 (38)	0.06
*Acceptability of systematic screening in pregnant women and practices*				
Do you think it is useful to systematically propose hepatitis B screening to pregnant women?				
Yes (a lot, somewhat, a little)	83 (95)	46 (98)	37 (92)	0.25
No (not at all, I do not know)	4 (5)	1 (2)	3 (8)	
In the last month, have you proposed hepatitis B screening to pregnant women?				
Yes (always, often)	42 (48)	32 (68)	10 (25)	< 10^−3^
No (rarely, never, I do not know)	45 (52)	15 (32)	30 (75)	

*HCW* Healthcare workers, *HBV* hepatitis B virus

^a^ Chi-square test or Fisher’s exact test

More than half (59%) of HCW reported that they had already benefited from training in HBV infection, and 67% in general counseling techniques, yet only 20% had received specific HBV counseling training. Furthermore, HCW in Group 2 were significantly more likely to have received training than HCW in Group 1, both in HBV infection (respectively, 72% versus 47%, *p* = 0.01) and in general counseling (respectively, 82% versus 54%, *p* = 0.01). Finally, 28% felt they were not adequately trained to correctly deliver counseling to HBV-positive patients.

#### HCW acceptability of systematic screening in pregnant women and perceived challenges to its implementation

HCW acceptability of systematic screening in pregnant women was high, with 95% of participants reporting that screening is useful to prevent MTCT (Table 5). This was confirmed in qualitative interviews.

- *“… It’s to protect the child, so it’s the only way to prevent the child from getting the disease.” (Interview HCW 9)*
- *“First, we need to know the mother’s status during antenatal consultations, [so we need to] do the analysis.” (Interview HCW 5)*

However, even though HCW favored systematic screening and counselling of pregnant women, only 68% of those in Group 1 and 25% in Group 2 (p < 10^− 3^) declared they had actually proposed such screening during the previous month (Table 5).

HCW mentioned several barriers to implementing systematic HBV screening in pregnant women. The first was that HBV testing was not free for pregnant women, unlike screening for other diseases, such as HIV and malaria. In addition, they mentioned that it is quite expensive, which can discourage some women. They suggested that HBV screening could be more effective in this resource-limited rural population if tests were free of charge.

- *“The prenatal checkup is 9,000 CFA (i.e. 15.7 USD)… the screening fees; it’s them [the pregnant women] that pay… You know, for HIV, you do not pay. If we could make hepatitis B screening free, that would be good for women and also for the population.” (Interview HCW 4)*

The second barrier described by HCW was the lack of availability of RDT in primary healthcare posts. Consequently, HCW have to refer pregnant women to the two district healthcare centers, which are located more than 5 km (i.e. approximately a one-hour walk) away for the majority of the population living in the districts of Fatick and Niakhar.

- *“Hepatitis B, I don’t have rapid tests, that’s to say that the women that come here, I send them to the laboratory because I don’t have the means to do screening and they get back to me with the results.” (Interview HCW 5)*

Finally, HCW mentioned their difficulties to communicate information about the disease to patients with a low educational level. Some HCW believed that less-educated patients do not have the skills to understand the risks and consequences of HBV.

- *“In contrast, people who didn’t go to school, well, you explain it to them as well as you can. Sometimes they do not even understand. I have received patients with viral hepatitis B but I am sure they do not know what it is. I explain, I explain it to them each time, but they’re not conscious of what it means.” (Interview HCW 3)*

### Vaccination at birth

#### Specific knowledge and skills

Almost all HCW (98%) correctly answered that vaccination at birth is a strategy for reducing perinatal transmission and 85% correctly answered that the first dose of HBV vaccine for children should be administered within 24 h after birth (Table 6). However, only 38% knew that three to four doses of HBV vaccine are required to ensure immunization in children, including a monodose at birth followed by two to three supplementary doses.

**Table 6:** Knowledge, skills and acceptability of HBV vaccination among healthcare workers participating in the quantitative component of the survey (*n* = 87)

Variable (% of missing data)	Total	HCW mainly involved in prenatal care activities (Group 1)	HCW only involved in vaccination activities (Group 2)	*p*-value^a^
	(*N* = 87)	(*N* = 47, 54%)	(*N* = 40, 46%)	
	N (%)			
*Specific knowledge and skills on vaccination*				
What are the potential strategies for reducing perinatal transmission?				
Vaccination at birth				
*Correct answer*	85 (98)	46 (98)	39 (97)	
*Incorrect answer*	2 (2)	1 (2)	1 (3)	0.71
When should the first dose of HBV vaccine be administered to children?				
*Correct answer*	74 (85)	40 (85)	34 (85)	0.99
*Incorrect answer*	13 (15)	7 (15)	6 (15)	
In total, how many injections are required to immunize children against hepatitis B infection?				
*Correct answer*	33 (38)	19 (40)	26 (65)	0.60
*Incorrect answer*	54 (62)	28 (60)	14 (35)	
*Acceptability of HBV vaccination at birth and practices*				
In your opinion, how effective is the hepatitis B vaccine? (1%)				
Very effective (> 95%) or moderately	80 (93)	43 (94)	37 (92)	0.59
Quite ineffective, I do not know	6 (7)	3 (6)	3 (8)	
Do you agree that the hepatitis B vaccine is safe?				
Strongly agree or rather agree	85 (98)	45 (96)	40 (100)	1
Neither agree or disagree, disagree (rather or strongly), I do not know	2 (2)	2 (4)	0	
Do you think it is useful to vaccinate newborns against hepatitis B within 24 h after birth?				
Yes (a lot, somewhat)	86 (99)	47 (100)	39 (97)	
A little, not at all, I do not know	1 (1)	0	1 (3)	0.45
In the past month, have you vaccinated newborns against hepatitis B within 24 h of their birth?				
Yes	62 (71)	32 (68)	30 (75)	0.48
No or I do not know	25 (29)	15 (32)	10 (25)	

*HCW* Healthcare workers, *HBV* hepatitis B virus

^a^ Chi-square test or Fisher’s exact test

#### HCW acceptability of HBV vaccination at birth and perceived challenges to its implementation

Overall, HCW were very confident about the efficacy of HBV vaccine: 93% declared it is effective and 98% said it is safe (Table 6). In addition, 99% agreed that vaccinating newborns within 24 h is useful and necessary. The high acceptability of the HBV vaccine was also highlighted during qualitative interviews.

- *“This really is a vaccine coming along at the right time, so it’s a really useful vaccine to prevent the disease. Especially when it is started right at birth. And we’ve seen that it’s a very accessible vaccine because it is free.” (Interview HCW 9)*

However, some HCW expressed concern about the effectiveness and safety of the birth dose for the babies born from a HBV-positive mother.

- *“At birth, we do it for all the babies. I wonder what the difference is between a baby whose mother is positive for HBV antibodies and one whose HBV antibody count is negative” (Interview HCW 6)*

HCW also reported that child vaccination at birth was well accepted by mothers.

- *“No, no, we don’t have problems really with them. There is no refusal. Because they’re also beginning to understand the usefulness and significance of these vaccines… we explain that this vaccine is to avoid this or that pathology and then they really understand.” (Interview HCW 7)*

Despite good acceptability of child vaccination at birth, only 75% of HCW in Group 2 and 68% in Group 1 declared they had actually vaccinated newborns in the month before the survey (Table 6).

HCW mentioned home deliveries as a barrier to vaccination at birth, as babies born at home are not always taken to healthcare facilities within the first 24 h of life for postnatal care.

- *“Now as part of prevention, giving the hepatitis vaccine at day zero, we need to sensitize women so that they give birth in the facilities, to limit home deliveries a little* ***.*** *” (Interview HCW 2)*
- *“When a home delivery happens, people that come 24 hours after it, we include them in our vaccination schedule. We put them into 2 categories. Children vaccinated in the first 24 hours, and those vaccinated after the first 24 hours.” (Interview HCW 7)*

HCW also reported that cold chain problems, including power outages or failures, posed a risk to vaccine efficacy and immunization activities.

- “*[…] It is the cold chain that sometimes is not up to scratch, because there are outages, the cold chain is faulty sometimes. If we had been equipped with solar fridges, because there’s always sun here, that could help.” (Interview HCW 4)*

Finally, they emphasized the difficulties associated with mothers’ compliance with their children’s vaccination schedule.

- *“It’s just that sometimes there are not educated, they forget the days of their appointments, they forget sometimes, but if we go to them to have them come, they come.” (Interview HCW 4)*

### Care and treatment

#### Specific knowledge

Overall, basic knowledge on HBV treatment was poor in HCW (Table 7). Only 45% correctly answered that effective treatment for chronic HBV exists, and only 38% knew that treatment for HBV is life-long. Moreover, only 10 and 7% knew that Tenofovir and Lamivudine, respectively, were effective treatments against HBV. In addition, the effectiveness of antiviral treatment in preventing perinatal transmission was not very well known: only 55% of HCW reported that this strategy was effective in reducing perinatal transmission.

**Table 7:** Knowledge of HBV care and treatment among healthcare workers participating in the quantitative component of the survey (*n* = 87)

Variables (% of missing data)	Total	HCW mainly involved in prenatal care activities (Group 1)	HCW involved exclusively in vaccination activities (Group 2)	*p*-value^a^
	(*N* = 87)	(*N* = 47, 54%)	(*N* = 40, 46%)	
	N (%)			
Is there an effective treatment against chronic hepatitis B infection? (2%)				
*Correct answer*	38 (45)	17 (37)	21 (54)	0.12
*Incorrect answer*	47(55)	29 (63)	18 (46)	
How long does antiviral therapy against chronic hepatitis B continue? (1%)				
*Correct answer*	33 (38)	21 (46)	12 (30)	0.12
*Incorrect answer*	55 (62)	25 (54)	28 (70)	
Which of the following treatments has proven efficacy against chronic hepatitis B infection?				
Lamiduvine (3%)				
*Correct answer*	6 (7)	4 (9)	2 (5)	0.41
*Incorrect answer*	78 (93)	41 (91)	37 (95)	
Tenofovir (5%)				
*Correct answer*	8 (10)	5 (11)	3 (8)	0.43
*Incorrect answer*	75 (90)	39 (89)	36 (92)	
What are the potential strategies for reducing perinatal transmission?				
Antiviral treatment for the mother (1%)				
*Correct answer*	47 (55)	28 (60)	19 (49)	0.21
*Incorrect answer*	39 (45)	19 (40)	20 (51)	
Immune globulin administration (2%)				
*Correct answer*	20 (23)	10 (21)	10 (26)	0.79
*Incorrect answer*	65 (77)	37 (79)	28 (74)	

*HCW* Healthcare workers, *HBV* hepatitis B virus

^a^ Chi-square test or Fisher’s exact test

Qualitative interviews further highlighted HCW lack of knowledge about HBV treatment, especially the existence of effective treatments to treat HBV.

*“There is no specific treatment for the disease” (Interview HCW 7)*

Some also believed that only symptomatic treatments are currently available.

*“Because you know that viral diseases are difficult to treat. So, for hepatitis there’s only symptomatic treatment because you cannot purify the blood as we say…” (Interview HCW 2)*

Finally, some HCW believed that treatment for hepatitis B only consisted of antibiotics, as was the case for HIV before the introduction of antiretroviral therapy.

*“… in principle, all the patients that come to us in the late stages, what the doctors do, they might take an antibiotic like Fleming, which is very powerful… by the way it’s a new molecule we [HCW] use… there’s Ciphran, Flagyl, Erytromycin, etc. those are very powerful antibiotics that we use” (Interview HCW 11)*

#### Practical challenges in relation to access to HBV care and treatment

HCW were aware of their lack of knowledge in management of HBV-infected individuals, explaining that they referred patients who screened HBV-positive (especially pregnant women) to specialists, and that their roles were limited to providing advice on diet and alcohol and tobacco consumption.

*“Really, I don’t know much. I send them to the doctor and if there is a treatment… I see they (doctors) ask for transaminases analyses and all that, to see how the liver is working. But I also ask women not to eat too many fatty foods, to avoid taking a lot of peanuts and stuff, in any case to avoid fatty foods.”* (*Interview HCW 5)*

*“So what we can tell people is to go see a specialist sometimes. At the same time, we give them advice on lifestyle habits, to avoid consuming certain foodstuffs and all that, such as alcohol, tobacco, to avoid them above all, because they can really intensify the disease.” (Interview HCW 7)*

HCW also highlighted the frequent use of traditional medicine as a first-line treatment, which delayed linkage to care. They indicated that HBV-positive patients only go for hospital consultation when their condition becomes critical with no possible improvement.

*“It’s that most of all which makes the patients come at a late stage, because as soon as they see the signs, they’ll go to see the traditional practitioner who is right beside them. And, as you know, that’s what’s going to aggravate the disease, with all that stuff they take… after the belly starts to bloat, the eyes, the kidneys are already wrecked and all that, it’s at this terminal stage they often come to hospital” (Interview HCW 3)*

*“It’s the first resort for hepatitis B, as I just said, the Serer [people] often say that maybe it’s a dietary problem, so they turn to traditional medicine” (Interview HCW 9)*

In addition, HCW mentioned a lack of access to HBV antiviral treatment, because of high cost and unavailability in most healthcare facility pharmacies, except for regional and national hospitals. Nevertheless, they indicated that in these hospitals, HBV antiviral treatment was provided free of charge only to HIV-HBV co-infected patients, and that HBV mono-infected patients did not have easy access to it.

*“Antivirals are not really accessible to everyone. This molecule is available only for patients living with HIV. These patients living with HIV who are infected with hepatitis B are treated. However, mono-infected patients are left out because they are not entitled to Tenofovir, which is intended for HIV patients. That is the problem. Because if you need to buy the molecule, it is expensive. Sometimes it is not available in pharmacies. That is the problem” (Interview HCW 3)*

Finally, in addition to the cost of drugs, HCW mentioned that HBV-positive patients must shoulder high costs of multiple medical examinations as part of their follow-up and treatment.

*“Often, when you follow the patients, with countless required check-ups, ultrasounds, hepatic check-ups to be done again and again, at a certain point that leads to a problem of financial means. So really, it is about a lack of means” (Interview HCW 1)*

*“These patients that we receive, the balance sheet is also extremely steep, the antibodies, all this to follow the evolution of the disease, are extremely expensive, they are not really accessible to all” (Interview HCW 3)*

## Discussion

Using a comprehensive mixed-methods approach, this is the first study to explore knowledge, perceptions, acceptability, barriers and levers related to HBV prevention strategies among HCW working at the decentralized level of the healthcare system in the rural region of Fatick in Senegal.

### Overview of the results

Three main findings emerged from the interviews with the HCW. First, HCW knowledge of key areas of HBV prevention and care - including transmission routes, screening options, prevention means and the existence of effective life-long treatment - seemed to be insufficient to provide adequate prevention and care interventions. This knowledge gap tended to be greater in HCW involved only in vaccination activities than in those involved mainly in prenatal care. Second, quantitative and qualitative data suggested high acceptability by HCW of systematic screening for HBV in pregnant women and HBV vaccination at birth in newborns, as well as positive perceptions about the efficacy and safety of vaccination. However, the implementation of these key interventions in routine care appeared to be suboptimal, as suggested by the significant proportion of HCW involved in prenatal care and/or vaccination activities who reported they had not performed either intervention during the previous month. Finally, our study highlighted some important barriers that may explain the suboptimal implementation of HBV prevention strategies and that may limit their effectiveness for preventing HBV MTCT. A significant proportion of HCW reported they had received no training on hepatitis B infection and/or on pre- and post-HBV test counseling. This is supported by the low level of knowledge observed in the survey participants. Other important barriers highlighted were the lack of available and free HBV RDT, the non-availability of antiviral treatment for pregnant women diagnosed HBV-positive, and missed opportunities for vaccination at birth because of home deliveries.

### HCW knowledge about HBV natural history, epidemiology, screening, vaccination and treatment

HCW lack of knowledge about HBV infection and its epidemiology is consistent with previous studies conducted in caregivers with similar profiles but working in urban health facilities in sub-Saharan Africa endemic areas, especially Ethiopia and Cameroon [14, 26]. In those studies, only approximately half of the HCW had a sound knowledge of hepatitis B infection. Furthermore, as in our study, the main transmission routes were not well known: a non-negligible proportion of HCW thought that HBV could be transmitted through the fecal-oral route (20% in Ethiopia and 44% in Cameroon) and did not know that HBV could be transmitted from mother to child (34% in Ethiopia). HCW knowledge on treatment for chronic HBV infection has been rarely investigated until now which is a reflection of the very limited access to and availability of HBV treatment in sub-Saharan Africa endemic areas. In a recent study conducted on Senegalese physicians working in the major hospitals of the two main cities of the country, 80% of the participants knew about the existence of an effective HBV treatment, 30% correctly answered that it is a life-long treatment, and 51 and 57%, respectively, identified lamivudine and tenofovir as HBV treatments [16]. These percentages were larger than those observed in our survey. However, ours did not include physicians.

Good knowledge of HBV infection, especially its epidemiology, transmission routes and treatment options, is essential to i) promote adherence to screening and vaccination schedule, ii) provide adequate counseling, and iii) refer positive cases to appropriate care. Our findings strongly suggest that inadequate training of HCW may hinder the effective implementation of HBV screening in pregnant women. Indeed, despite good acceptability of this key strategy, HCW felt insufficiently trained to deliver adequate counseling to HBV-positive patients. A study in Burkina Faso highlighted that poor HBV knowledge among HCW is a major barrier to linkage to HBV care [25]. Although HCW at decentralized levels are not responsible for HBV care or treatment in Senegal, they constitute the gateway to the specialist care currently delivered in major hospitals in the country. They therefore play an important role at the district level in screening cases, particularly pregnant women, and in linkage to care of HBV-positive individuals. Our results suggest there is an urgent need to improve HBV-related capacity building in HCW working at the decentralized level of the healthcare system, as they are the first line of healthcare for the population, especially in low-resource, rural and underserved areas.

### Challenges faced by HCW to implementing prevention activities and policy recommendations

In our study, HCW reported a number of other barriers that may explain the suboptimal implementation of HBV prevention activities, including the high cost of screening and lack of RDTs in primary health posts. This is especially true for pregnant women, the consequence being that they need to travel to a healthcare facility equipped with a laboratory for HBV screening. Although HBV testing is included in prenatal tests prescribed during pregnancy, it is not free (unlike for HIV or malaria) and costs approximately 5.26 USD. In addition, the full prenatal check-up - including blood count, blood group, glycaemia and Hbs antigen testing - costs 15.78 USD. Consequently, pregnant women do not always perform all the tests prescribed. This means that those who are HBV-positive may not be aware they are infected and risk transmitting HBV to their baby. The provision of free HBV RDT for pregnant women at the decentralized level is indispensable to ensure early diagnosis and in turn prevent HBV transmission. With regard to the implementation of vaccination at birth, our results are consistent with the WHO data which estimated that, in 2017, only 50% of newborns in Senegal received HBV vaccine at birth [27]. The home birth rate there, estimated at 22%, is a potential barrier to achieving high vaccination rates at birth [28]. Simple community interventions, such as the use of community health volunteers to inform health facilities of all home births and more effective interpersonal communication as recommended by WHO, could improve the current situation [29].

### Study limitations

Some limitations of our study need to be acknowledged. First, self-reports of systematic screening and vaccination at birth can be affected by social desirability bias. HCW may have overemphasized the importance of screening pregnant women for HBV and of vaccinating newborns. However, the mixed-methods approach used here enabled us to investigate HCW perceptions on HBV prevention strategies in depth. It also helped us to understand challenges faced by HCW in the implementation of MTCT prevention activities which may explain the suboptimal implementation of these strategies [30]. Moreover, the mixed-methods approach is known to increase the reliability of data collected when the research topic is sensitive in nature, as may have been the case here: in the quantitative component of the survey, HCW may have seen the study as an examination of their knowledge [31].

Second, we used a two-stage reasoned sampling approach and not a random sampling method for the quantitative component. However, we believed the risk of selection bias is limited for the following reasons: i) the sample of healthcare facilities, selected based on a purposive approach, was quite representative of the diversity of the healthcare supply in the two study districts and in the Fatick region (see Additional file 1); ii) participation in the survey was proposed to all eligible HCW in the study area; and iii) 84% of eligible HCW finally participated in the survey.

Third, the relatively small sample size may have limited statistical power to detect significant inter-group differences in knowledge about HBV, acceptability and perceptions of prevention and care strategies. However, it is comparable with the sample size of similar surveys conducted among HCW in sub-Saharan Africa [16, 32]. Moreover, the present study was conducted in two districts of the region of Fatick, which account for 37 of all 110 healthcare facilities in the region (i.e. 34%) and 274 of 597 (46%) HCW. Although the study results cannot be generalized for the whole of Senegal, as they may not be representative of other districts and regions, they do provide quite a good picture of HCW knowledge, perceptions and challenges related to the implementation of HBV prevention and care activities in the Fatick region. Our study is particularly relevant to Senegal, as Fatick has been selected by the national hepatitis program as a pilot region for the decentralization of HBV care. The work here will also provide a good basis for further exploration of issues related to decentralization in other sub-Saharan African countries.

## Conclusions

Despite the study’s limitations, our findings may inform public-health decision-making on HBV prevention in rural Senegal. We found that most HCW working in decentralized health facilities do not have sufficient knowledge about HBV to enable them to carry out HBV prevention activities effectively, especially regarding prevention of MTCT. Similarly, despite the high level of acceptability of systematic screening for pregnant women and vaccination at childbirth by HCW, several structural barriers hamper the implementation of these strategies. There is an urgent need for health authorities to strengthen HBV prevention and care activities at the decentralized level of the Senegalese health care system. First, HCW must be trained up on the key areas of HBV infection, especially transmission routes, prevention means and treatment options. Second, access to HBV screening for pregnant women needs to be facilitated by providing RDT free of charge and making HBV treatment available for HBV-positive mothers. Finally, effective community-based interventions should be implemented to identify homebirths and guarantee timely vaccination.

## Additional files

Additional file 1: Main characteristics of healthcare facilities: i) in the Fatick region (*N* = 110), ii) in the two selected districts of Fatick and Niakhar (*N* = 37) and iii) in the study sample (*N* = 15). Compares the main characteristics of the healthcare facilities selected in the study sample with that of the healthcare facilities of the Fatick and Niakhar districts and of the Fatick region. (DOCX 16 kb)

Additional file 2: Quantitative questionnaire. Presents the questionnaire which has been developed for the quantitative component of the study. (DOCX 24 kb)

## Data Availability

The datasets used and/or analyzed during the current study are available from the corresponding author on reasonable request.

## Abbreviations

EPI: Expanded Immunization Program
HBV: Hepatitis B Virus
HCW: Healthcare workers
HIV: Human Immunodeficiency Virus
IQR: Interquartile Range
MD: Missing data
MTCT: Mother-To-Child Transmission
RDT: Rapid Diagnostic Test
WHO: World Health Organization
