# Supplementary material for: Prevention and care of hepatitis B in the rural region of Fatick in Senegal: a healthcare workers’ perspective using a mixed methods approach
Source: BMC Health Serv Res. 2019 Sep 4;19:627. doi: 10.1186/s12913-019-4416-3 (PMC6727484; doi:10.1186/s12913-019-4416-3)
Supplement: Supplementary file 1 — Main characteristics of healthcare facilities: i) in the Fatick region (N = 110), ii) in the two selected districts of Fatick and Niakhar (N = 37) and iii) in the study sample (N = 15). Compares the main characteristics of the healthcare facilities selected in the study sample with that of the healthcare facilities of the Fatick and Niakhar districts and of the Fatick region. (DOCX 16 kb) [file 12913_2019_4416_MOESM1_ESM.docx]

Additional Table 1: Main characteristics of healthcare facilities: i) in the Fatick region (N=110), ii) in the two selected districts of Fatick and Niakhar (N=37) and iii) in the study sample (N=15). The additional Table 1 compares the main characteristics of the healthcare facilities selected in the study sample with that of the healthcare facilities of the Fatick and Niakhar districts and of the Fatick region.

| **Healthcare facility characteristics** | **Sample (N=15)^1^** | **Fatick and Niakhar districts (N=37)** | | **Fatick region**  **(N=110)** |
| --- | --- | --- | --- | --- |
|  | N (%) | | | |
| Location:  - urban  - rural | 4 (27%)  11 (73%) | | 4 (11%)  33 (89%) | 7 (6%)  103 (94%) |
| Status:  - public  - private | 13 (87%)  2 (13%) | | 32 (87%)  5 (13%) | 91 (83%)  19 (17%) |
| Number of healthcare workers in the healthcare facility:  - <10  - ≥10 | 12 (80%)  3 (20%) | | 35 (95%)  2 (5%) | 102 (93%)  8 (7%) |
| Healthcare facility category:  - Hospital  - Healthcare center  - Healthcare post | 1 (7%)  2 (13%)  12 (80%) | | 1 (3%)  2 (5%)  34 (92%) | 1 (1%)  7 (6%)  102 (93%) |

^1^ Sample of 15 healthcare facilities (including the Fatick regional hospital) selected using a purposive approach to represent the diversity of the healthcare supply in the two districts of Niakhar and Fatick, and in the region of Fatick.
