# Supplementary material for: Prevention and care of hepatitis B in the rural region of Fatick in Senegal: a healthcare workers’ perspective using a mixed methods approach
Source: BMC Health Serv Res. 2019 Sep 4;19:627. doi: 10.1186/s12913-019-4416-3 (PMC6727484; doi:10.1186/s12913-019-4416-3)
Supplement: Supplementary file 2 — Quantitative questionnaire. Presents the questionnaire which has been developed for the quantitative component of the study. (DOCX 24 kb) [file 12913_2019_4416_MOESM2_ESM.docx]

**Additional file 2: Quantitative questionnaire (translated from the original french version).**

**The additional file 2 presents the questionnaire, which has been developed for the quantitative component of the study.**

**Questionnaire _AMBASS ANRS 12356 Project** - « Study on the hepatitis B knowledge, perceptions and practices in caregivers working in the rural area of Niakhar in the region of Fatick, in Senegal »

**Participant identification number: ……………………………………………………………………………**

**Name of the healthcare facility: ……………………………………………………………………………….**

**Decentralization level of the healthcare facility:**

□ Regional hospital □ District healthcare center □ Primary healthcare post

| **Questions** | | **Possible answers** |
| --- | --- | --- |
| ***Socio-demographic characteristics*** | | |
| **What is your birthday?** | **\|__\|__\| \|__\|__\| \|__\|__\|__\|__\|**  Day Month Year | |
| **What is your gender?** | Female | |
|  | Male | |
| **What is your level of education?** | Never went to school | |
|  | Primary | |
|  | Middle school | |
|  | Secondary school | |
|  | Higher | |
|  | Koranic school | |
|  | Other | |
| **What is your initial training in the field of health or social care?** | Nurses (registered and licensed professional) | |
|  | Nursing assistant | |
|  | Midwife | |
|  | Laboratory technician | |
|  | Community healthcare worker | |
| **Since when do you practice your profession?** | **\|__\|__\| \|__\|__\| \|__\|__\|__\|__\|**  Day Month Year | |
| ***Training, experience and activities in the field of HBV*** | | |
| **Have you already benefited from training in HBV?** | Yes (in my initial training) | |
|  | Yes (in continued education) | |
|  | No | |
|  | I do not know | |
| **Have you already had training in counseling or therapeutic education for other diseases?** | Yes | |
|  | No | |
|  | I do not know | |
| **Have you already had training in counseling or therapeutic education for hepatitis B?** | Yes | |
|  | No | |
|  | I do not know | |
| **Do you feel you are trained well enough to provide adequate counseling to HBV-positive patients?** | Yes, a lot | |
|  | Yes, somewhat | |
|  | Yes, a little | |
|  | Not at all | |
|  | I do not know) | |
| **In which activities are you involved in your routine work practice?** | Prenatal care *(yes; no; I do not know)* | |
|  | Post-natal care *(yes; no; I do not know)* | |
|  | Deliveries *(yes; no; I do not know)* | |
|  | Vaccination *(yes; no; I do not know)* | |

| **Are you involved in the following activities on hepatitis B?** | Vaccination at birth (<72 hours) *(yes; no; I do not know)* | |
| --- | --- | --- |
|  | Vaccination in newborns (>72h) and children *(yes; no; I do not know)* | |
|  | Counseling to pregnant women *(yes; no; I do not know)* | |
|  | Prescription of screening to pregnant women *(yes; no; I do not know)* | |
| **In the last month, have you proposed screening for hepatitis B to pregnant women?** | Yes, always | |
|  | Yes, often | |
|  | Rarely | |
|  | Never | |
|  | I do not know | |
| **In the last month, have you vaccinated newborns against hepatitis B** **within 24h of their birth?** | Yes | |
|  | No | |
|  | I do not know | |
| ***General knowledge of HBV*** *(natural history and epidemiology)* | | |
| **What kind of infectious agent causes hepatitis B?** | | Virus |
|  |  | Bacterium |
|  |  | I do not know |
| **What organ is affected by HBV?** | | Liver |
|  |  | Lung |
|  |  | Stomach |
|  |  | Heart |
|  |  | I do not know |
| **What complications can be caused by hepatitis B?** | | Liver cancer *(yes; no; I do not know)* |
|  |  | Cirrhosis *(yes; no; I do not know)* |
| **What are the modes of HBV transmission?** | | Dust (yes; no; I do not know) |
|  |  | Contaminated water *(yes; no; I do not know)* |
|  |  | Perinatal transmission (from mother to child) *(yes; no; I do not know)* |
|  |  | Breastfeeding *(yes; no; I do not know)* |
|  |  | Horizontal transmission (during childhood through contacts with infected blood) *(yes; no; I do not know)* |
|  |  | Percutaneous transmission or transmission through mucosae *(yes; no; I do not know)* |
|  |  | Sexual transmission *(yes; no; I do not know)* |
| **Is perinatal transmission (from mother-to-child) one of the main modes of transmission in Senegal?** | | Yes |
|  |  | No |
|  |  | I do not know |
| **Are people infected by HBV during childhood (<1 year) going to develop chronic infection?** | | Yes, more than 80% of them |
|  |  | Yes, approximately 50 % of them |
|  |  | Yes, only 5 % |
|  |  | I do not know |
| ***Specific knowledge on HBV screening*** | | |
| **What are the priority groups for which routine screening of chronic hepatitis B is recommended?** | | Pregnant women *(yes; no; I do not know)* |
|  |  | Blood donors *(yes; no; I do not know)* |
|  |  | Health Care Workers *(yes; no; I do not know)* |
|  |  | HIV-positive patients *(yes; no; I do not know)* |
| **What kind of test can be used to screen for HBV in adults and children >1year** | | Rapid diagnostic test *(yes; no; I do not know)* |
|  |  | Laboratory-based immunoassay (yes; no; I do not know) |
| ***Specific knowledge on HBV vaccination*** | | |
| **What are the potential strategies for reducing perinatal transmission?** | | Vaccination at birth *(yes; no; I do not know)* |
|  |  | Antiviral treatment for the mother *(yes; no; I do not know)* |
|  |  | Immune globulin administration *(yes; no; I do not know)* |
| **When should the first dose of HBV vaccine be administered to children?** | | Within 24 hours after birth |
|  |  | Within the first week of life |
|  |  | At 6 weeks |
|  |  | At one year old |
|  |  | I do not know |
| **In total, how many injections are required to immunize children against hepatitis B infection?** | | 1 injection |
|  |  | 2 injections |
|  |  | 3 injections |
|  |  | 4 injections |
|  |  | Other |
|  |  | I do not know |
| ***Specific knowledge on HBV treatment*** | | |
| **Is there an effective treatment against chronic hepatitis B infection?** | | Yes |
|  |  | No |
|  |  | I do not know |
| **Which of the following treatments has proven efficacy against chronic hepatitis B infection?** | | Lamivudine *(yes; no; I do not know)* |
|  |  | Tenofovir *(yes; no; I do not know)* |
|  |  | Traditional treatment *(yes; no; I do not know)* |
| **How long does antiviral therapy against chronic hepatitis B infection continue?** | | One week |
|  |  | A month |
|  |  | For life |
|  |  | I do not know |
| ***Acceptability and perception of MTCT prevention strategies*** | | |
| **Do you think it is useful to systematically propose hepatitis B screening to pregnant women?** | | Yes, a lot |
|  |  | Yes, somewhat |
|  |  | Yes, a little |
|  |  | Not at all |
|  |  | I do not know |
| **In your opinion, how effective is the hepatitis B vaccine?** | | Very effective (>95%) |
|  |  | Moderately effective |
|  |  | Quite ineffective |
|  |  | I do not know |
| **Do you agree that the hepatitis B vaccine is safe?** | | Strongly agree |
|  |  | Rather agree |
|  |  | Neither agree or disagree |
|  |  | Rather disagree |
|  |  | Strongly disagree |
|  |  | I do not know |
| **Do you think it is useful to vaccinate newborns against hepatitis B within 24h after birth?** | | Yes, a lot |
|  |  | Yes, somewhat |
|  |  | Yes, a little |
|  |  | Not at all |
|  |  | I do not know |

HBV: hepatitis B virus.
